# Supplementary material for: Antiseptic Effects and Biosafety of a Controlled-Flow Electrolyzed Acid Solution Involve Electrochemical Properties, Rather than Free Radical Presence
Source: Microorganisms. 2022 Mar 30;10(4):745. doi: 10.3390/microorganisms10040745 (PMC9032035; doi:10.3390/microorganisms10040745)
Supplement: Supplementary file 1 [file microorganisms-10-00745-s001.zip › microorganisms-1603705-supplementary.pdf]

Table S1. Antimicrobial profile of bacteria strains.

	Gram-positive		Gram-negative				
**Antibiotics**	***S. aureus***	***E. faecalis***	***E. coli***	***K. pneumoniae***	***E. cloacae***	***A. baumannii***	***P. aeruginosa***
Oxacillin	R	ND	ND	ND	ND	ND	ND
Ampicillin	R	R	R	R	S	ND	ND
Piperacillin/tazobactam	ND	ND	ND	ND	ND	ND	S
Vancomycin	S	S	ND	ND	ND	ND	ND
Ceftriaxone	ND	ND	R	S	S	R	R
Ceftazidime	ND	ND	R	S	S	R	R
Cefepime	ND	ND	R	S	S	R	R
Imipenem	ND	ND	S	S	S	R	S
Meropenem	ND	ND	S	S	S	R	S
Ertapenem	ND	ND	S	S	S	ND	ND
Aztreonam	ND	ND	R	S	S	R	R
Gentamicin	S	ND	R	R	S	S	R
Amikacin	ND	ND	S	R	S	R	R
Ciprofloxacin	R	S	R	S	S	S	R
Levofloxacin	R	S	R	S	S	S	R
Trimethoprim/sulfamethoxazole	S	ND	R	ND	ND	S	S
Rifampicin	S	ND	ND	ND	ND	ND	ND
Colistin	ND	ND	S	S	S	S	S
Erythromycin	S	S	ND	ND	ND	ND	ND
Clindamycin	S	S	ND	ND	ND	ND	ND

Susceptibility (S), Resistance (R) and Not Determined (ND).

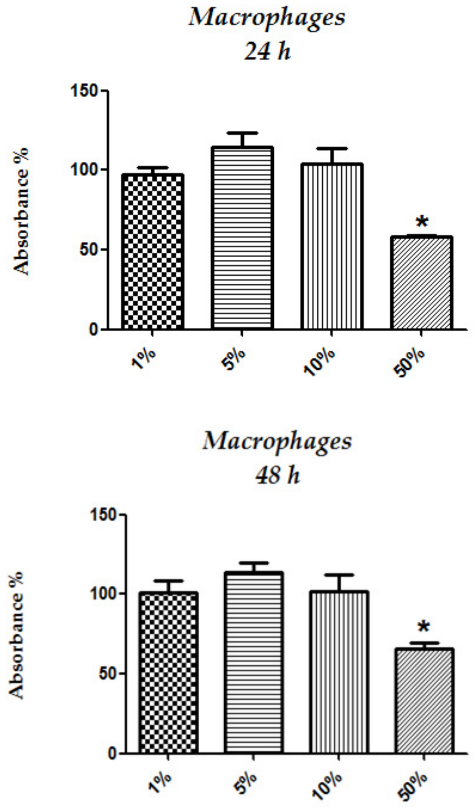

Figure S1. Proliferation assay of macrophages. BrdU uptake was evaluated in macrophage cultures treated with 1--50% CFEAS at 24 and 48 h. No statistically significant changes in proliferation were observed at any culture condition when compared with untreated controls.

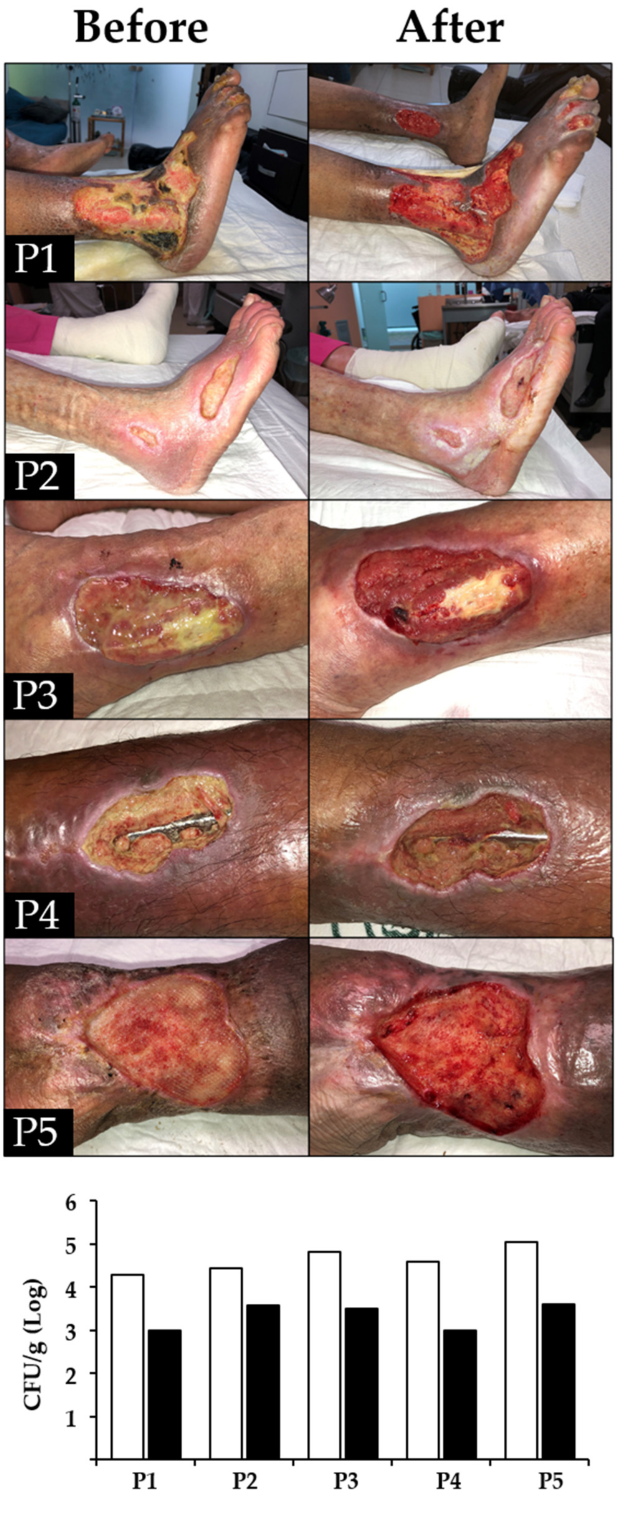

Figure S2. Effects of treatment with CFEAS of several human infected subacute and chronic wounds. Clinical pictures from five patients (P1–P5) with subacute or chronic

wounds. Left and right panels show pictures from patients with wounds before and after treatment with CFEAS, respectively. Plot represents the log of CFU/g of tissue from every patient.
